# Cost of childbirth in Upper West Region of Ghana: a cross-sectional study

**DOI:** 10.1186/s12884-022-04947-x

**Published:** 2022-08-04

**Authors:** Maxwell A. Dalaba, Paul Welaga, Mustapha Immurana, Martin Ayanore, Justina Ane, Laata L. Danchaka, Chieko Matsubara

**Affiliations:** 1 Institute of Health Research, University of Health and Allied Sciences, Box 31, Ho, Ghana; 2 Social Science Department, Navrongo Health Research Centre, Box 114, Navrongo, Ghana; 3 C.K. Tedam University of Technology and Applied Sciences, Box 24, Navrongo, Ghana; 4 School of Public Health, University of Health and Allied Sciences, Box 31, Ho, Ghana; 5 University of Environment and Sustainable Development, PMB Somanya, Ghana; 6 Community Development Alliance, Box 490, Wa, Ghana; 7 Bureau of International Medical Cooperation, National Centre for Global Health and Medicine, Toyama 1-21-1, Tokyo, Japan

**Keywords:** Access, Cost of delivery, Childbirth, Free maternal health policy, Upper West Region, Ghana

## Abstract

**Background:**

Out-of-pocket payment (OOPP) is reported to be a major barrier to seeking maternal health care especially among the poor and can expose households to a risk of catastrophic expenditure and impoverishment.This study examined the OOPPs women made during childbirth in the Upper West region of Ghana.

**Methods:**

We carried out a cross-sectional study and interviewed women who gave birth between January 2013 and December 2017. Data on socio-demographic characteristics, place of childbirth, as well as direct cost (medical and non-medical) were collected from respondents. The costs of childbirth were estimated from the patient perspective. Logistics regression was used to assess the factors associated with catastrophic payments cost. All analyses were done using STATA 16.0.

**Results:**

Out of the 574 women interviewed, about 71% (406/574) reported OOPPs on their childbirth. The overall average direct medical and non-medical expenditure women made on childbirth was USD 7.5. Cost of drugs (USD 8.0) and informal payments (UDD 5.7) were the main cost drivers for medical and non-medical costs respectively. Women who were enrolled into the National Health Insurance Scheme (NHIS) spent a little less (USD 7.5) than the uninsured women (USD 7.9). Also, household childbirth expenditure increased from primary health facilities level (community-based health planning and services compound = USD7.2; health centre = USD 6.0) to secondary health facilities level (hospital = USD11.0); while home childbirth was USD 4.8. Overall, at a 10% threshold, 21% of the respondents incurred catastrophic health expenditure. Regression analysis showed that place of childbirth and household wealth were statistically significant factors associated with catastrophic payment.

**Conclusions:**

The costs of childbirth were considerably high with a fifth of households spending more than one-tenth of their monthly income on childbirth and therefore faced the risk of catastrophic payments and impoverishment. Given the positive effect of NHIS on cost of childbirth, there is a need to intensify efforts to improve enrolment to reduce direct medical costs as well as sensitization and monitoring to reduce informal payment. Also, the identified factors that influence cost of childbirth should be considered in strategies to reduce cost of childbirth.

## Background

Even though a number of global and national initiatives have been established to improve access to maternal health care, the progress made so far has been slow. For instance, in 2017, about 295,000 women died during pregnancy and childbirth [1]. The vast majority of these deaths (94%) occurred in low-resource settings, and most could have been prevented [1].

Access to affordable and quality health care services remains a challenge in many low-income countries, particularly in Sub-Saharan Africa. Many preventable maternal deaths still occur across most developing countries partly due to the financial hardship women face in seeking health care [2]. Out-of-pocket payment (OOPP) is reported to be a major barrier to seeking maternal health care especially among the poor and can expose households to a risk of catastrophic expenditure and impoverishment [3, 4]. It has been reported in previous studies that pregnant women made OOPPs of GHC 17.50/USD 8.60 during childbirth in Ghana [5], USD 28.76 in rural Zambia [6] and as high as $93.3 per child childbirth in India [7].

In Ghana, to address some of the challenges in seeking health care and to attain universal health coverage (UHC), the government implemented interventions such as the free maternal health care policy through the national Health Insurance Scheme (NHIS) [8] and expansion of primary health care services such as the community-based health planning and services (CHPS) [9].

Although previous studies have reported the positive effect of free maternal health programmes on the utilization of maternal health care [4, 10, 11], but OOPP still existed as women paid considerable amounts of money to seek maternal care [3, 11, 12]. However, there are limited studies on the effects of the Ghana’s maternal health interventions (free maternal health policy and CHPS) on the cost of childbirth care [12].

It is therefore uncertain as to whether despite these government interventions, OOPPs associated with seeking maternal health services continue to exit, and if so, at what level. This study therefore examined the OOPPs women made durin childbirth in the Upper West Region of Ghana.

## Methods

### Study area

The study was conducted in the Upper West Region (UWR), Ghana. The UWR is one of the 16 regions of Ghana, located in the northern part of the country. Its population is 901,502 (2021), and has low population density [13]. This region is bordered by Burkina Faso to its north and west. Subsistence agriculture is the mainstay of the population. The region is one of the poorest in Ghana. The major ethnic groups in the region are the Dagaaba, Sissala, and Wala [13]. There are a total of 242 health facilities providing various types of services in the UWR. These include 3 district government hospitals, 1 regional hospital, 2 Christian Health Association of Ghana (CHAG) hospitals and 3 private hospitals. The rest include 5 polyclinics, 66 health centres, 10 clinics and 147 CHPS compounds and 4 maternity homes [13].

### Study design

The study design was cross-sectional, and a quantitative approach was used to collect data between January and April 2018. A structured questionnaire was administered to women of reproductive age who gave birth between January 2013 and December 2017. If a mother had multiple childbirth during the period, cost of childbirth was collected on the most recent childbirth. The structured questionnaire had sections on socio-demographic characteristics, place of childbirth, cost of childbirth and household wealth.

### Sample size

The sample size for this study was calculated by assuming that 50% of the women of reproductive age in the study districts would have given birth between 2013 and 2017 [14–16]. Using a 95% confidence level and a design effect of 1.3 [12] to account for the clustering effect in the districts would require a sample size of 500. The sample size required was calculated using the formula:

$$n=\frac{{DEFF\times (z)}^{2}\times p\times (1-p)}{{(d)}^{2}}$$

where *n* = sample size, z = desired level of confidence at 95% (standard value of 1.96), *p* = Proportion of childbirth within 5 years (0.5), d = level of precision(0.05), and DEFF = estimated design effect (1.3).

$$A\;sample\;size\left(n\right)=\frac{{1.3(1.96)}^2\times0.778\times(1-0.778)}{{(0.05)}^2}$$

$$n=500$$

Adjusting for a non-response rate of 15% ((0.15*500) + 500) resulted in a sample size of 575.

### Sampling and data collection

The study’s respondents were chosen using a multistage sampling approach. To begin, we divided the UWR’s 11 districts into three ethnic groups: i) Dagaati districts (Daffiama Bussie Issa (DBI), Jirapa, Lambussie, Lawra, Nadowli, and Nandom); ii) Wala districts (Wa East, Wa Municipality, and Wa West); and iii) Sissala districts (Sissala East and Sissala West).

Secondly, one district from each ethnic group was randomly selected. This strategy ensured a geographic and ethnic representation across the region. Accordingly, Nadowli district was selected to represent the Dagaati ethnic group, Wa West district was selected to represent the Wali ethnic group and Sissala East to represent the Sissala ethnic group.

Thirdly, using a random approach, we chose a sub-district within each district, and then 5 communities inside each sub-district. After that, interviewers were assigned to the communities and were given the task of identifying 39 respondents per community by moving from one household to the next until they reached their sample size. Households were randomly selected in each community by the interviewer by spinning a pen in the centre of the community. The direction in which the pen was pointing was followed, and the first house was chosen from there. If a household had more than one woman with children under the age of 5 years ( gave birth between 2013 and 2017), only one of them was chosen at random for an interview. After that, the interviewer moves on to the next house/household. This was repeated until the interviewer had obtained the maximum number of respondents for the community. Thus, respondents were women of reproductive age who gave birth between January 2013 and December 2017. Thus, respondents were women of reproductive age who gave birth between January 2013 and December 2017.

Graduate-level data collectors were recruited for data collection. The selection criteria were based on understanding and speaking the local language(s), familiarity with the environment, and previous experience with data collection. Data collectors were trained on the research protocol and the questionnaire and guidelines for conducting interviews. A pre-test was conducted during the training to determine the appropriateness of the questions in the questionnaire and all errors were rectified before the final data collection.

### Data processing and analysis

The data were collected using a paper-based questionnaire and then double entered by two data entry clerks using EPI Data 6.1. After entry, the data was cleaned and verified and inconsistent entries from the two data entry clerks were checked from the source questionnaire and corrections made.

Data were then transferred to STATA version 16.0 for cleaning and analysis. Frequencies and cross tabulations were used to clean up the data by finding outliers, missing values, and verifying for consistency among variables. Tables were created to display frequencies, proportions and means. The cost of childbirth was estimated from the patient perspective.

The total childbirth cost was calculated as the sum of direct medical and non-medical costs. The direct medical costs covered OOPPs for registration cards, consultations, diagnosis (scanning and laboratory tests), drugs and medical supplies. Direct non-medical costs included OOPPs for transportation to and from health facilities, informal payments as well as other non-medical costs such as disinfectant, a rubber bed spread, cotton, gauze, childbirth mat and spirit. Informal payments are payments in cash or non-cash that, in addition to the specified contribution, are provided by patients or their relatives to the health care providers for services that the patients are legally entitled to receive [17].

The annual income of households was calculated using the estimated cost of yields or proceeds from agricultural products such as crops and poultry as well as from salaries or wages from businesses, and income from investments or gifts.

Furthermore, a wealth assets index was estimated using principal component analysis (PCA). The household assets used in computing the wealth index include main materials used for the floor, materials used for the roof, materials used for exterior walls, the main sources of drinking water, and ownership or use of a toilet facility, radios, clocks, television sets, mobile phones, landline telephones, refrigerators, freezers, electric generators, washing machines, computers, photo cameras, digital versatile discs (DVDs), sewing machines, beds, table cabinets/cupboards, wrist watches, bicycles, motorcycles, animal-drawn carts, cars and boats. Households were then assigned to five quintiles which described household wealth levels and represented as: poorest (Q1), very poor (Q2), poor (Q3), less poor (Q4) and least poor (Q5).

Catastrophic heath expenditure for childbirth was calculated. Catastrophic payments occur when total OOPPs for health care exceeds a certain threshold of a household’s resources (income or expenditure) usually using a threshold between 5 and 40% [8, 18, 19]. In this study, we used a threshold of 10% as used in other studies in other African countries [19, 20]. Thus, catastrophic payment was said to occur when the childbirth cost was ≥ 10% of the household monthly income.

We also investigated the impact of household childbirth expenditure on poverty using the 2017 Ghana national monthly poverty line of GHC 110/USD24.4 [21]. We estimated the poverty headcount ratio, also called the poverty incidence, to measure the proportion of households living below the monthly poverty line before and after childbirth costs as used in other studies [22].

To determine the factors that influence household catastrophic payment for childbirth, a logistics regression was used [6]. The outcome variable was catastrophic payment (binary variable, 1 = incurred catastrophic payment; 0 = did not incur catastrophic payment) and the independent variables were occupation, education, insurance status, wealth index and place of childbirth.

All costs in the study were collected in Ghana Cedis (GHC), and the results are presented in USD using the average exchange rate of 2017 (USD 1 = GHC 4.5) [23].

### Ethical consideration

The Navrongo Health Research Centre Institutional Review Board (Approval ID: NHRCIRB232) and the National Centre for Global Health and Medicine (NCGM), Japan(Approval ID: NCGM-G-0020510–00), provided ethical approval The study process was explained to all of the respondents, and written informed consent was obtained. Illiterate respondents consented in their preferred local language, with those who agreed to participate having their thumbs printed on the consent form.

## Results

### Background characteristics of respondents

Table 1 presents the background characteristics of respondents. The total number of respondents included in the analysis were 574. The average age of the respondents was 29 years, and 75.8% were between 20 and 34 years old. About 61% of the respondents had never been to school, 19.9% completed primary school and 5.8% had secondary or higher education. On occupation, most of the respondents were farmers/traders (90.4%). About 85% of respondents had enrolled into the NHIS at the time of interview. The average household monthly income was estimated at USD 205.9 with Standard deviation (SD) of USD662.8.

**Table 1:** Background characteristics of respondents

Variable	Number (*n* = 574)	Percent (%)
Age group		
15–19	23	4.0
20–34	435	75.8
35 +	116	20.2
Ethnicity		
Wala	98	17.1
Sissala	204	35.5
Dagaaba	242	42.2
Other	30	5.2
Education		
Never Been to School	349	60.8
Primary School	114	19.9
Junior High/Middle/Technical School	78	13.6
Secondary School and higher	33	5.8
Occupation		
Farmer/trader	519	90.4
Artisan/Public worker	32	5.6
Unemployed	23	4.0
Insurance status (NHIS)		
Insured	487	84.8
Not insured	87	15.2
Average monthly household income	USD205.9(SD:USD662.8)	

### Childbirth cost

Table 2 shows the costs incurred by respondents during childbirth. Out of the 574 respondents, about 71% (406/574) made OOPPs for childbirth, of which about 15% (62/406) were not insured (not enrolled into the NIHS) and 85% (344/406) were insured (enrolled into the NIHS).

**Table 2:** Childbirth cost (USD)

**Variables**	**Cost Category**		**Insured**	**Uninsured**	**Total Average Cost (SD)**
		**Number (n)**	**Mean (SD)**	**Mean (SD)**	
Medical	Registration card	82	0.8 (0.7)	0.9(0.5)	0.8(0.7)
	Consultation	16	4.1(1.9)	1.7(1.4)	2.9(2.1)
	Diagnosis	43	3.1(4.2)	3.7(6.6)	3.2(4.8)
	Drugs	89	7.6(15.3)	9.2(13.4)	8.0(14.9)
	Medical supplies	101	4.3(3.8)	3.1(3.2)	4.2(3.8)
	Total	213	6.0(11.8)	8.9(11.8)	6.5(11.8)
Non-medical	Transportation	288	4.1(8.6)	2.8(3.3)	3.9(8.1)
	Informal payments	22	6.9(11.2)	1.6(1.2)	5.7(10.0)
	Non- medical payments such disinfectant, rubber bed spread, cotton, gauze, spirit	120	3.5(2.5)	4.1(3.6)	3.6(2.7)
	Total	344	5.1(8.9)	3.8(4.9)	4.9(8.3)
Overall		406	7.5(13.3)	7.9(11.4)	7.5(13.1)

About 52% (213/406) of the respondents made OOPPs on direct medical costs, such as the cost of a registration card, consultation, diagnosis, drugs and medical supplies during the last childbirth at an average cost of USD 6.5. The women who were enrolled into the NHIS spent less (USD 6) on direct medical costs compared to the uninsured women (USD 8.9). Expenditure on drugs accounted for the largest direct medical cost (USD 8).

The average non-medical cost was USD 4.9, and the insured spent more (USD 5.1) than the uninsured women (USD 3.8). On average, women spent USD 3.9 on transportation to travel to and from a health facility to give birth, and the travel time was 47 min. Informal payment accounted for the largest direct non-medical cost (USD5.7); and the expenditure on informal payments was more among the insured (USD 6.9) than the uninsured (USD 1.6).

Overall, the average OOPPs made on direct and non-direct medical costs was USD 7.5. Women who were enrolled into the NHIS spent a little less (USD 7.5) than the uninsured women (USD 7.9) on overall cost of childbirth. About 55% of the total cost of childbirth was on direct non-medical cost and 45.1% was on direct medical cost.

### Childbirth cost by socioeconomic status (wealth quintile) and place of childbirth

Table 3 shows the average childbirth cost by socioeconomic status of respondents and place of childbirth. We found evidence of varying childbirth costs across socioeconomic status. For instance, while women from the poorest households (quintile 1) spent an average of USD 9.8 on child childbirth, their counterparts in the richest/least poor households (quintile 5) spent an average of USD 8.7 on childbirth.

**Table 3:** Total childbirth cost by socioeconomic status and place of childbirth (USD)

Wealth Quintile	Mean(SD)
Quintile 1 (Poorest)	9.8(15.0))
Quintile 2 (Very poor)	6.3(7.6)
Quintile 3 (Poor)	4.9(5.9)
Quintile 4 (Less poor)	8.0(18.0)
Quintile 5 (Least poor)	8.7(14.4)
Total	7.5(13.1)
**Place of** childbirth	
Home	4.8(3.7)
CHPS Compound	7.2(17.3)
Health Centre	6.0(11.5)
Hospital	11.0(12.4)
Total	7.5(13.1)

*SD* Standard Deviation

Also, household childbirth expenditure increased from lower levels (primary level) to higher levels (secondary level) health facilities. Whereas the average household’s expenditure on childbirth at a CHPS compound was USD7.2, it was USD 6.0 at a Health Centre, USD 11.0 at a hospital and USD 4.8 when the child was given birth at home (Table 3).

### Financial burden imposed by childbirth cost across wealth quintiles

About 21% of respondents’ households spent more than 10% of their monthly income on childbirth and were deemed to have made catastrophic payments. When catastrophic payments were disaggregated by wealth quintiles, the results showed that women in poorest households incurred higher catastrophic payment (24.6%) compared to women in least poor households (14%). This shows a higher catastrophic health expenditure in the poorest households than the richest households.

Considering the impoverishing effects of childbirth expenditures, the findings revealed that 16.9% of the respondents were living below the poverty line before childbirth expenditure. After the expenditure on childbirth, the proportion of households below the poverty line increased to 18.5%. Thus, 1.6% of respondents’ households were further entrenched in poverty (Table 4).

**Table 4:** Catastrophic payment and poverty incidence due to childbirth expenditure (%)

Wealth Quintile	Catastrophic Payment	Poverty Head Count (Before childbirth Cost)	Poverty Head Count (after childbirth Cost)	Net Count
Quintile 1 (Poorest)	24.6	29.8	30.4	0.6
Quintile 2 (Very poor)	31.9	28.3	30.4	2.1
Quintile 3 (Poor)	13.0	8.7	9.6	0.9
Quintile 4 (Less poor)	21.2	12.4	14.8	2.4
Quintile 5 (Least poor)	14.0	4.4	7.9	3.5
Total	20.9	16.9	18.5	1.6

Monthly Poverty line = Ghc110/USD24.4

### Predictors of catastrophic payment due to child childbirth cost

The results of the regression analysis showed that place of childbirth and household wealth were statistically significant factors associated with catastrophic payments (Table 5).

**Table 5:** Logistic regression of predictors of catastrophic payment due to child childbirth cost

Catastrophic payments	Odds Ratio	Standard Error	*p* > Value	95% Confidence Interval
Home (B)				
CHPS Compound	3.99	2.08	0.01***	1.44,11.08
Health Centre	5.62	2.78	0.00***	2.13,14.84
Hospital	13.78	6.99	0.00***	5.10,37.27
***Insurance status***				
Not insured (B)				
Insured	1.02	0.31	0.94	0.56,1.87
***Wealth index***				
Quintile 1 (Poorest)(B)				
Quintile 2 (Very poor)	1.34	0.43	0.35	0.72,2.50
Quintile 3 (Poor)	0.43	0.16	0.02**	0.20,0.89
Quintile 4 (Less poor)	0.78	0.27	0.47	0.40,1.53
Quintile 5 (Least poor)	0.38	0.15	0.01***	0.18,0.82
***Occupation***				
Unemployed (B)				
Farmer/trader	0.54	0.27	0.22	0.20,1.44
Artisan	0.71	0.47	0.60	0.19,2.59
***Educational status***				
Never been to school (B)				
Primary school	0.67	0.20	0.19	0.37,1.22
Junior High/Middle/Technical School	0.96	0.33	0.90	0.49,1.87
Secondary and higher	1.77	0.88	0.26	0.66,4.70

^*^ *p* < 0.1, ** *p* < 0.05, *** *p* < 0.01; B represents the base category

As compared to women who gave birth at home, women who gave birth at a CHPS compound, Health centre, and Hospital had 4.99 times higher odds (*p* < 0.01), 5.61 times higher odds (*p* < 0.01), and 13.78 times higher odds (*p* < 0.01) of incurring catastrophic payment due to cost of childbirth, respectively. Women who were from a poor household (quintile 3) had 0.43 times lower odds (*p* < 0.05) of incurring catastrophic payment relative to women from poorest households (quintile 1). Also, women who were from least poor household (quintile 5) had 0.38 times lower odds (*p* < 0.01) of incurring catastrophic payment relative to women from poorest households (quintile 1).

## Discussion

The study examined the OOPPs women made during child childbirth in the UWR of Ghana. The study results revealed that women in the UWR incurred some costs during child childbirth (USD7.5). Despite the free maternal health care policy which was implemented through the NHIS in 2008, the results showed that about 85% of insured women made OOPPs for direct and non-direct medical costs and spent an average of USD 7.5. With the free maternal health policy, pregnant women are not supposed to pay for any direct medical costs as the policy aims to facilitate access to free and quality maternal health care services such as antenatal, childbirth and postnatal care services at health care facilities. The benefit package includes: consultations, laboratory investigations, X-rays, ultrasound scanning and prescription medicines on the NHIS drugs list [24, 25]. However, our findings showed that insured women incurred direct medical costs (USD 6.0). The reasons for OOPPs for direct medical supplies were due to the non-availability of medicines, laboratory reagents and ultrasound machines at the health facilities, and the fact that women are asked to get these items/services at private facilities at a cost. The study results corroborate findings from a similar study in northern Ghana where pregnant women made OOPPs (GHC 17.50/USD 8.60) during childbirth despite the free maternal health policy [5]. In addition, despite free maternal policy in Zambia, average cost of childbirth was reported to be as much as USD 28.76 in rural Zambia [6] as well as $93.3 in India [7]. That notwithstanding, in our study, women who were enrolled into the NHIS /free maternal policy spent a little less (USD 7.5) than the uninsured women (USD 7.9), and therefore is the need to improve pregnant women enrolment onto the policy.

The burden of OOPP on childbirth is worrisome, particularly in poor households, as our study revealed that women from the poorest households spent more than relatively wealthy households. Additionally, a considerable proportion of households (21%) of respondents spent more than 10% of their monthly income on childbirth and therefore faced catastrophic payments, while 1.6% were pushed below the national poverty line by the cost of childbirth. Household expenditure on childbirth can run down a household’s resources, especially poor households, and thereby limit the household’s capacity to obtain basic needs which would worsen its poverty status [26]. The most recent living standards survey in the country reported that the UWR is the poorest region in the country and seven out of every ten in the region are poor (70.7%) [13]. This indicates the burden households face regarding childbirth cost. It is therefore important to intensify efforts to reduce childbirth costs in the region.

Expenditure on direct non-medical costs incurred by women during childbirth was quite substantial (USD 4.9). Some of these non-medical items such as disinfectant, rubber bed spread, cotton, gauze, childbirth mat and spirit are supposed to be covered by the free maternal health policy, but amazingly women (both insured and uninsured) were asked to buy them OOPP. This highlights the challenges to implementing the free maternal health policy. Our findings are consistent with similar observations from a study in Zambia which reported that women spent on childbirth supplies, such as disinfectant, gloves, and cord clamps [6].

Surprisingly, informal payment was high and represented the main non-medical cost driver (USD 5.7). In a typical Ghanaian setting, households are usually happy when they have a safe childbirth and are sometimes willing to make informal payments such as presenting gifts to the health workers. Sometimes too, the health workers themselves solicit these informal payments from the women. A previous study reported informal payments in 91.8% of pregnant women seeking maternal care in India at a cost of US$0.03–101 [27]. Also, over 31% and 3.6% of the mothers in the Public and Private Not for Profit Hospitals (PNFP) respectively made informal payments during childbirth and the average payments given in the Public Hospital was Uganda Shillings (UGX) 65,750 while that in the PNFP Hospital was UGX11,000 [28]. These informal payments are a financial barrier to seeking maternal care, particularly poor households, and therefore efforts are needed to monitor health workers as well as sensitize community members to curd the situation.

Cost of childbirth varied by childbirth locations, and expectedly, more was spent at the secondary level facility (hospital) than at the primary health facility level (health Centre and CHPS). Regression analysis also showed that place of childbirth was an important factor that contributed to childbirth costs. As women move from lower level health facilities to higher level facilities, they incur higher childbirth costs. Women who gave birth at CHPS compound, Health centre, and Hospital had over 3 times higher odds of incurring catastrophic payments due to cost of childbirth, relative to women who gave birth at home. Similar finding have been reported were in comparison to women who gave birth at home, women who gave birth at a primary health centre had over four times the odds of spending anything on their childbirth [6]. Furthermore this study reported lower home childbirth costs (USD 4.8) compared to the Zambian study that reported as much as USD21.82 for cost of home childbirth [6]. This highlights the importance of functional primary health facilities, particularly CHPS compounds were midwives are allocated to CHPS compounds to provide child birth services to community members. In addition, on average, women from the middle-class households(poor/quintile 3) had a lower odd to incur catastrophic cost compared to women from the poorest households (quintile 1) reflecting the severity of the burden of cost of childbirth to poorest households which can worsen their poverty situation.

Transportation costs hinder access to maternal health care at health facilities and increases the risk of maternal deaths [29]. This study showed that non-medical costs such as transportation cost was USD3.9. Though Ghana has made progress regarding the expansion of primary health facilities such as CHPS compounds and the introduction of midwives to the CHPS compounds to provide maternal care including skilled childbirth, more efforts is still needed to improve access to health care and reduce the cost of transportation. Also, more efforts are still needed to reduce distance or transport costs to health facilities through the building of more health facilities in hard to reach areas. Moreover, to make the health facilities, particularly the CHPS compounds more accessible and functional, roads in the rural areas should be improved, given that bad roads result in increased transport costs, delays in reaching a health facility as well as delays in moving from the lower health facility to a referral facility.

## Limitations of the study

A possible limitation of this study is recall bias, given that respondents were asked to recall expenditure over a 5-year period. The reported expenditure could either be overestimated or underestimated, especially because respondents did not show receipts on expenditure but only verbal reporting of expenditure. However, the probing techniques used may minimize this bias, and we do not expect this bias to adversely affect the study results.

In addition, the study did not assess the costs disaggregated by spontaneous childbirth and caesarean childbirth, since the emphasis was just on the cost of childbirth. Nevertheless, we acknowledge that this is a limitation, given that there could be cost variations between these forms of childbirth. Indirect cost (productivity lost due to childbirth) was not included in the analysis. We acknowledge that this as a limitation which could have increased the cost of childbirth.

Another limitation of this study is that it relied exclusively on a quantitative approach. Including a qualitative approach (mixed method) would have complemented the quantitative data and therefore provided rich data for the study. However, the quantitative instrument was well developed and detailed, capturing all important components that are relevant to the study. We do not expect the exclusion of the qualitative approach to negatively affect the study results.

## Conclusions

Women incurred considerable costs during childbirth with 21% of households spending more than one-tenth of their monthly income on childbirth. These households faced the risk of catastrophic payments and impoverishment. Childbirth expenditure by the insured women was a little lower than the uninsured suggesting some positive effect of the NHIS. It is to improve the enrolment of women into the NHIS. Though some progress has been made in Ghana regarding the expansion of primary health care, more efforts are still needed to improve access to health care and reduce the cost of transportation. Interventions to improve access to health care in hard-to-reach areas will contribute to reducing non-medical costs which are mainly travel related costs. Informal payments during childbirth are common and can be a financial strain to households. Other background factors such as place of childbirth and wealth that influence cost of childbirth should be considered in government strategies to reduce cost of childbirth. Furthermore, appropriate resources must be made available at health institutions to prevent women from paying for medical supplies and services outside of NHIS-accredited health facilities.

## Data Availability

Relevant data and materials based on which conclusions were made are included in the manuscript. However, the corresponding author can be contacted if someone wants data from the study.

## Abbreviations

CHO: Community health officers
CHPS: Community-based health planning and services
DBI: Daffiama Bussie Issa
NHIS: National health insurance
OOPP: Out-of-pocket payment
PCA: Principal component analysis
UHC: Universal health coverage
UWR: Upper West Region
